# Salusin-β induces foam cell formation and monocyte adhesion in human vascular smooth muscle cells via miR155/NOX2/NFκB pathway

**DOI:** 10.1038/srep23596

**Published:** 2016-03-23

**Authors:** Hai-Jian Sun, Ming-Xia Zhao, Tong-Yan Liu, Xing-Sheng Ren, Qi Chen, Yue-Hua Li, Yu-Ming Kang, Guo-Qing Zhu

**Affiliations:** 1 Key Laboratory of Cardiovascular Disease and Molecular Intervention, Department of Physiology, Nanjing Medical University, Nanjing, Jiangsu 210029, China; 2 Department of Pathophysiology, Nanjing Medical University, Nanjing, Jiangsu 210029, China; 3 Department of Physiology and Pathophysiology, Cardiovascular Research Center, Xi’an Jiaotong University School of Medicine, Xi’an 710061, China

## Abstract

Vascular smooth muscle cells (VSMCs) are indispensible components in foam cell formation. Salusin-β is a stimulator in the progression of atherosclerosis. Here, we showed that salusin-β increased foam cell formation evidenced by accumulation of lipid droplets and intracellular cholesterol content, and promoted monocyte adhesion in human VSMCs. Salusin-β increased the expressions and activity of acyl coenzyme A:cholesterol acyltransferase-1 (ACAT-1) and vascular cell adhesion molecule-1 (VCAM-1) in VSMCs. Silencing of ACAT-1 abolished the salusin-β-induced lipid accumulation, and silencing of VCAM-1 prevented the salusin-β-induced monocyte adhesion in VSMCs. Salusin-β caused p65-NFκB nuclear translocation and increased p65 occupancy at the ACAT-1 and VCAM-1 promoter. Inhibition of NFκB with Bay 11-7082 prevented the salusin-β-induced ACAT-1 and VCAM-1 upregulation, foam cell formation and monocyte adhesion in VSMCs. Scavenging ROS, inhibiting NADPH oxidase or knockdown of NOX2 abolished the effects of salusin-β on ACAT-1 and VCAM-1 expressions, p65-NFκB nuclear translocation, lipid accumulation and monocyte adhesion in VSMCs. Salusin-β increased miR155 expression, and knockdown of miR155 prevented the effects of salusin-β on ACAT-1 and VCAM-1 expressions, p65-NFκB nuclear translocation, lipid accumulation, monocyte adhesion and ROS production in VSMCs. These results indicate that salusin-β induces foam formation and monocyte adhesion via miR155/NOX2/NFκB-mediated ACAT-1 and VCAM-1 expressions in VSMCs.

Atherosclerosis is positively associated with the formation of foam cells characterized by cholesterol esters and triglycerides accumulation in the cytoplasm^1^,^2^. The foam cell formation is a critical step in the atherosclerosis and plays essential role in the destabilization, rupture and erosion of atherosclerotic plaque^3^. Most of foam cells have been found to be derived from macrophages, but VSMCs give rise to a considerable number of foam cells as well^4^. Simultaneous thymidine autoradiography and immunostaining with cell type-specific monoclonal antibodies showed that about 30% of the labeled cells were macrophages and 45% were VSMCs in advanced atherosclerosis lesions in both Watanabe heritable hyperlipidemic rabbits and hypercholesterolemic fat-fed rabbits^5^. On the other hand, leukocyte recruitment from blood stream to the intima of vessels is a crucial event in the pathogenesis of atherosclerosis and a potential candidate for therapeutic approaches of atherosclerosis^6^. Cell adhesion molecules including intercellular adhesion molecule-1 (ICAM-1) and vascular cell adhesion molecule-1 (VCAM-1) are important for the initiating event of leukocyte recruitment to vascular endothelium and VSMCs in the development of atherosclerosis^7^. Although several adhesive mechanisms have been provided to be responsible for leukocyte recruitment in atherosclerosis, the mechanisms for the foam cell formation from VSMCs and the leukocyte recruitment in atherosclerosis are poorly elucidated.

Salusin-β is a peptide with 20 amino acids, which is translated from an alternatively spliced mRNA of TOR2A, a gene encoding a protein of the torsion dystonia family^8^. The initial 18 amino acids of human salusin-β have high homology with the N-terminal sequence of rat salusin^9^. Salusin-β is widely expressed within vasculature and central nervous system^10^. Our previous studies have showed that central salusin-β is involved in sympathetic activation and hypertension in hypertensive rats^11^,^12^,^13^, and peripheral salusin-β contributes to vascular remodeling associated with hypertension by promoting human VSMC proliferation via cAMP-PKA-EGFR-CREB/ERK pathway and vascular fibrosis via TGF-β1-Smad pathway^14^. Salusin-β is dominantly detected within VSMCs and fibroblasts in the human coronary atherosclerotic plaques^15^. Atherosclerotic lesions are deteriorated in apolipoprotein E-deficient mice with chronic salusin-β infusion^16^. Plasma salusin-β levels in subjects with diabetes mellitus, coronary artery disease, and cerebrovascular disease showed distinctly higher levels than healthy controls, and it may contribute to the pathogenesis of atherosclerosis^17^. However, the roles of salusin-β in the foam formation and monocyte adhesion in human VSMCs are rarely known. Thus, fully elucidating the roles of salusin-β and its underlying mechanisms in the foam cell formation and monocyte adhesion in VSMCs may be useful for the prevention and treatment of vascular lesions in atherosclerosis.

## Results

### Effects of salusin-β on foam cell formation and monocyte recruitment

**Figure 1:**
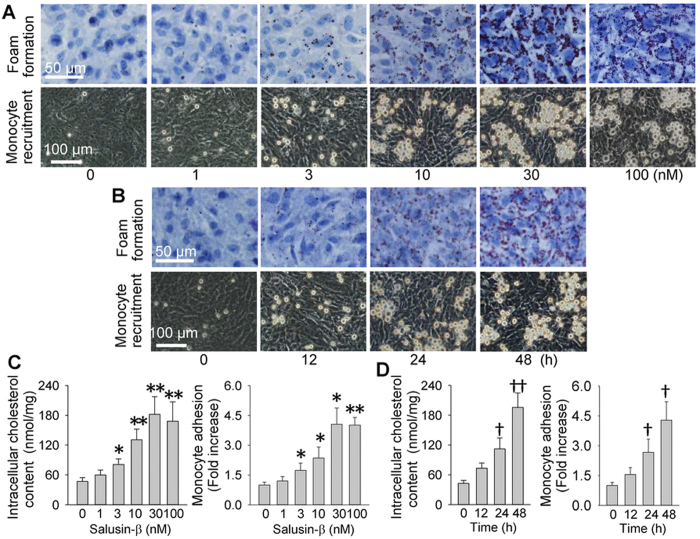
Effects of salusin-β on the foam formation and monocyte recruitment in VSMCs. (**A**) representative images showing the dose effects of salusin-β (1, 3, 10, 30 or 100 nM for 48 h) on the foam formation and monocyte recruitment; (**B**) representative images showing the time effects of salusin-β (30 nM for 0, 12, 24 or 48 h) on foam formation and monocyte recruitment; (**C**) dose effects of salusin-β (1, 3, 10, 30 or 100 nM for 48 h) on intracellular cholesterol content and monocyte adhesion; (**D**) time effects of salusin-β (30 nM for 0, 12, 24 or 48 h) on intracellular cholesterol content and monocyte adhesion. Values are mean ± S.E.M. ^*^P < 0.05 and ^**^P < 0.01 vs. 0 nM, ^†^P < 0.05 and ^††^P < 0.01 vs. 0 h. n = 6 for each group.

Salusin-β dose- and time-relatedly increased the accumulation of lipid droplets in VSMCs and the adhesion of monocyte to VSMCs, and the maximal effects were observed at the dose of 30 nM of salusin-β for 48 h (Fig. 1A,B). Total intracellular cholesterol quantification and the number of adherent cells further confirmed that salusin-β stimulated VSMC foam cell formation and monocyte recruitment (Fig. 1C,D).

### Effects of salusin-β on ACAT-1 and VCAM-1

**Figure 2:**
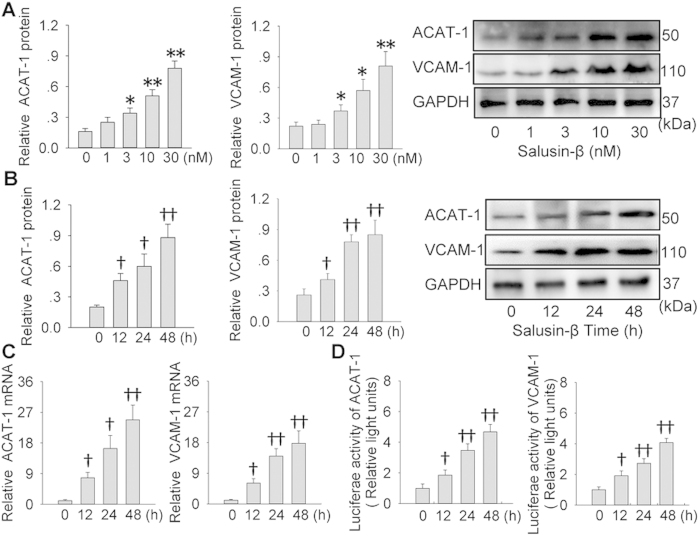
Effects of salusin-β on the expressions and promoter activity of ACAT-1 and VCAM-1 in VSMCs. (**A**) dose effects of salusin-β (0, 1, 3, 10 or 30 nM for 48 h) on ACAT-1 and VCAM-1 protein expressions. (**B**) time effects of salusin-β (30 nM for 0, 12, 24 or 48 h) on ACAT-1 and VCAM-1 protein expressions. (**C**) effects of salusin-β (30 nM) on ACAT-1 and VCAM-1 mRNA expressions of ACAT-1 and VCAM-1 proteins for indicated time. (**D**) effects of salusin-β (30 nM) on promoter activity of ACAT-1 and VCAM-1 for indicated time. Values are mean ± S.E.M. ^*^P < 0.05 and ^**^P < 0.01 vs. 0 nM; ^†^P < 0.05 and ^††^P < 0.01 vs. 0 h. n = 4 for each group in (**A**–**C**); n = 6 for each group in (**D**).

It is known that ACAT-1 promotes foam cell formation by promoting intracellular cholesteryl ester synthesis^18^, and VCAM-1 are an important cell adhesion molecule for leukocyte recruitment in the development of atherosclerosis^7^. Salusin-β upregulated the ACAT-1 and VCAM-1 protein levels in VSMCs in concentration- and time-dependent manner, and the maximal effects were found at the concentration of 30 nM of salusin-β for 48 h (Fig. 2A,B). Furthermore, it increased ACAT-1 and VCAM-1 mRNA levels, which were parallel to those of protein expressions (Fig. 2C). Luciferase reporter genes assay showed that salusin-β enhanced the promoter activities in the ACAT-1 and VCAM-1 truncated forms containing proximal NF-κB binding sites (Fig. 2D).

### ACAT-1 or VCAM-1 siRNA retards effects of salusin-β

**Figure 3:**
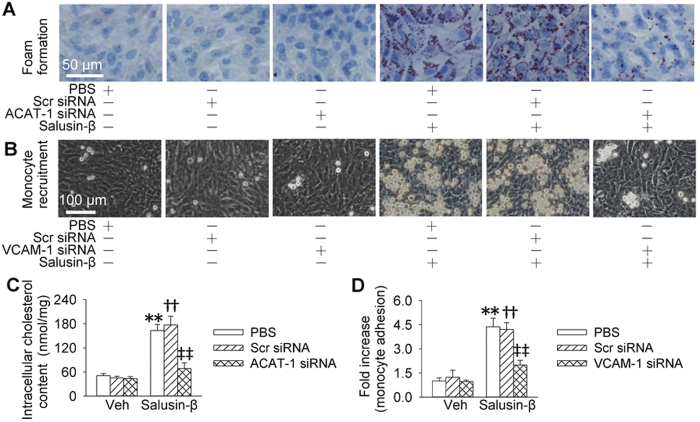
Effects of knockdown of ACAT-1 and VCAM-1 on foam formation and monocyte recruitment response to salusin-β in VSMCs. VSMCs were transfected with ACAT-1 or VCAM-1 siRNA or scrambled siRNA (100 nM) for 24 h, and then treated with or without salusin-β (30 nM) for 48 h. (**A**) effects of ACAT-1 siRNA on salusin-β-induced foam formation. (**B**) effects of VCAM-1 siRNA on monocyte recruitment response to salusin-β. (**C**) effects of ACAT-1 siRNA on the intracellular cholesterol content in response to salusin-β. (**D**) effects of VCAM-1 siRNA on salusin-β-induced monocyte adhesion. Values are mean ± S.E.M. ^*^P < 0.05 and ^**^P < 0.01 vs. PBS. ^†^P < 0.05 and ^††^P < 0.01 vs. Scr siRNA. ^‡^P < 0.05 and ^‡‡^P < 0.01 vs. Veh (vehicle). n = 6 for each group in (**C**) n = 4 for other groups.

To determine the importance of ACAT-1 in salusin-β-induced foam cell formation or VCAM-1 in salusin-β-induced monocyte recruitment, the effects of ACAT-1 siRNA and VCAM-1 siRNA were examined. Knockdown of ACAT-1 impeded salusin-β-induced accumulation of lipid droplets and increase in total intracellular cholesterol contents in VSMCs (Fig. 3A,C), but had no significant effects on salusin-β-induced monocyte adhesion in VSMCs (Fig. S1). Knockdown of VCAM-1 prevented salusin-β-induced monocyte adhesion in VSMCs (Fig. 3B,D), but had no significant effects on salusin-β-induced accumulation of lipid droplets and increase in total intracellular cholesterol contents in VSMCs (Fig. S2). The knockdown efficiency was confirmed by the reduced protein expression in VSMCs (Fig. S3). Moreover, ACAT-1 overexpression promoted foam formation in VSMCs (Fig. S4), and VCAM-1 overexpression increased the adhesion of monocyte to VSMCs in VSMCs (Fig. S5). The efficiency of overexpression was confirmed by the increased protein expressions in VSMCs (Fig. S6).

### NF-κB activation contributes to effects of salusin-β

**Figure 4:**
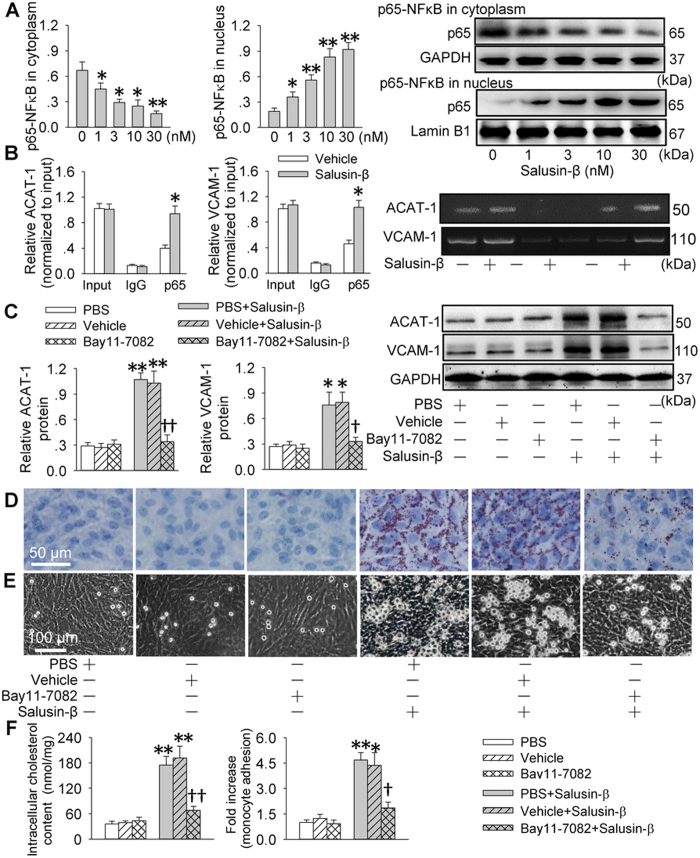
NFκB-p65 nuclear translocation involved in ACAT-1 and VCAM-1 transcriptions response to salusin-β in VSMCs. (**A**) effects of different doses of salusin-β (1, 3, 10 or 30 nM) on NFκB-p65 nuclear translocation. (**B**) ChIP analysis was performed with the indicated antibodies, and qPCR was underwent with a synthesized DNA fragment containing the NFκB element in human ACAT**-**1 gene promoter or VCAM-1 promoter. (**C)** effects of a NFκB inhibitor Bay11-7082 on salusin-β-induced ACAT-1 and VCAM-1 expressions. (**D**) effects of Bay11-7082 on salusin-β-induced foam formation. (**E**) effects of Bay11-7082 on monocyte adhesion response to salusin-β. (**F**) effects of Bay11-7082 on intracellular cholesterol content and monocyte adhesion. VSMCs were incubated with Bay11-7082 for 6 h and then treated with or without salusin-β (30 nM) for 48 h. Values are mean ± S.E.M. ^*^P < 0.05 and ^**^P < 0.01 vs. 0 nM, Vehicle or PBS. ^†^P < 0.05 and ^††^P < 0.01 vs. PBS+Salusin-β or Vehicle+Salusin-β; n = 4 for each group.

Salusin-β elicited an increase in nuclear p65-NFκB levels and a reduction in cytoplasmic p65-NFB levels in VSMCs in dose-related manner (Fig. 4A) and time-related manner (Fig. S7). As expected, salusin-β increased the binding of NF-κB to ACAT-1 and VCAM-1 promoter in VSMCs (Fig. 4B). Inhibition of NFκB with Bay11-7082 repressed the NFκB activity and nuclear NFκB level response to salusin-β in VSMCs (Fig. S8). The up-regulated ACAT-1 and VCAM-1 expressions in response to salusin-β were abrogated by inhibition of NFκB with Bay11-7082 (Fig. 4C). Pretreatment with Bay11-7082 inhibited the salusin-β-induced foam cell formation and adhesion of monocyte to VSMCs (Fig. 4D,E).

### NOX2-derived ROS mediates the effects of salusin-β

**Figure 5:**
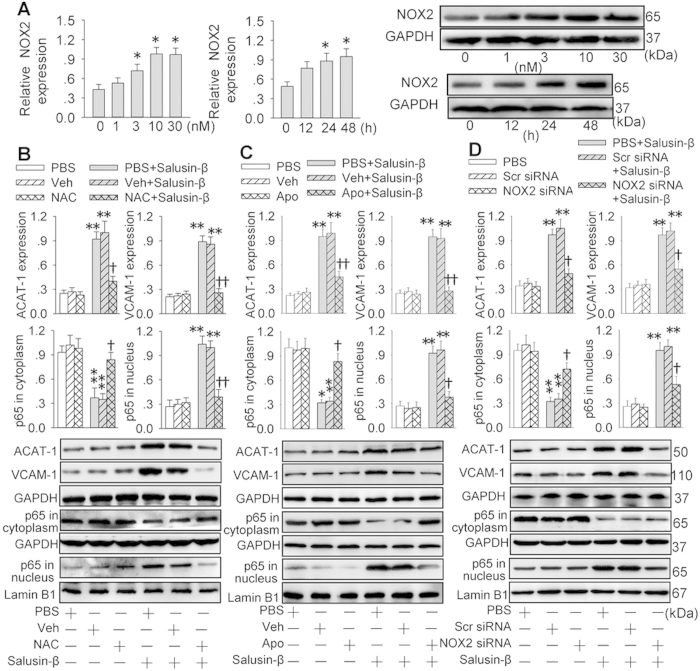
Nicotinamide-adenine dinucleotide phosphate Oxidase 2 (NOX2) derived reactive oxygen species (ROS) contributed to p65-NFκB nuclear translocation and ACAT-1 and VCAM-1 transcriptions response to salusin-β in VSMCs. (**A**) dose and time effects of salusin-β on the NOX2 expression. (**B**) effects of a free-radical scavenger *N*-acetylcysteine (NAC, 1 mM) on ACAT-1 and VCAM-1 expressions and p65-NFκB nuclear translocation. (**C**) effects of a NAD(P)H oxidase inhibitor apocynin (Apo, 100 μM) on ACAT-1 and VCAM-1 expressions and p65-NFκB nuclear translocation. The VSMCs were pretreated with NAC or Apo for 30 min and then challenged with salusin-β (30 nM) for 48 h. (**D**) effects of NOX2 siRNA (100 nM) on ACAT-1 and VCAM-1 expressions and p65-NFκB nuclear translocation. VSMCs were pretreated with NOX2 siRNA for 24 h and then stimulated with salusin-β (30 nM) for 48 h. Values are mean ± S.E.M. ^*^P < 0.05 and ^**^P < 0.01 vs. 0 h, 0 nM or PBS or Veh; ^†^P < 0.05 and ^††^P < 0.01 vs. PBS+Salusin-β or Veh+Salusin-β. n = 4 for each group.

**Figure 6:**
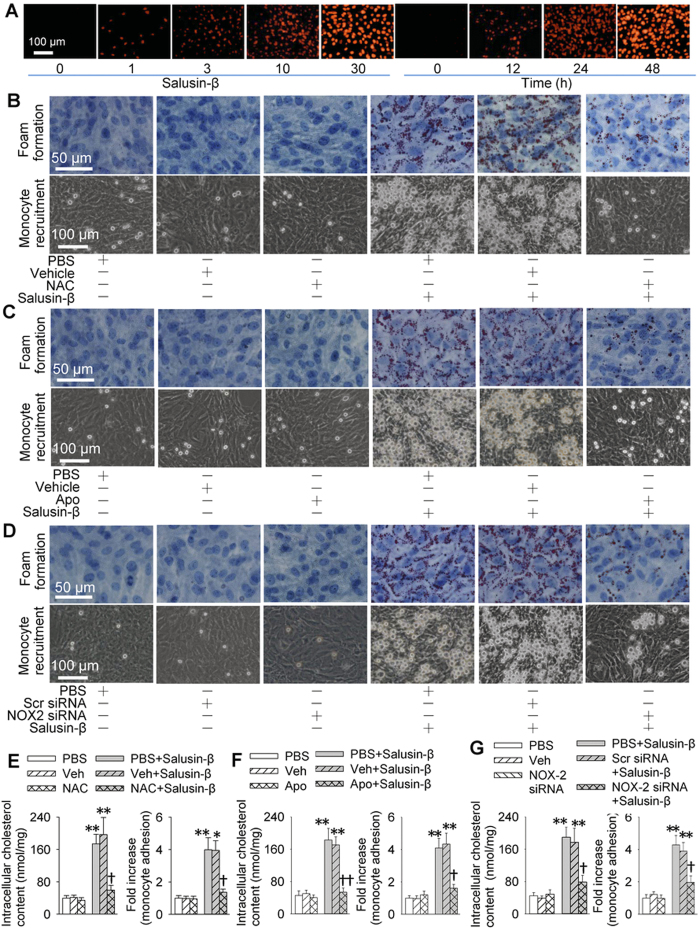
Effects of salusin-β on ROS production and effects of NAC, apocynin and NOX2 siRNA on foam formation, monocyte adhesion and intracellular cholesterol content in VSMCs. (**A**) dose-effects (0, 1, 3, 10 and 30 nM for 48 h) and time-effects (30 nM for 0, 12, 24 and 48 h of salusin-β on ROS production determined with DHE fluorescent probe. (**B–D**) effects of a free-radical scavenger NAC (1 mM), a NADPH oxidase inhibitor apocynin (Apo, 100 μM) and NOX2 siRNA (100 nM) on foam formation and monocyte recruitment. (**E–G**), effects of NAC, Apo and NOX2 siRNA on intracellular cholesterol content and monocyte adhesion. Values are mean ± S.E.M. ^*^P < 0.05 and ^**^P < 0.01 vs. PBS or Veh; ^†^P < 0.05 and ^††^P < 0.01 vs. PBS+Salusin-β or Veh+Salusin-β. n = 4 for each group.

Incubation of VSMCs with salusin-β dose- and time- relatedly increased NADPH oxidase 2 (NOX2) expressions (Fig. 5A) and ROS production (Fig. 6A). Salusin-β-induced ACAT-1 and VCAM-1 expressions, and p65-NFκB nuclear translocation in VSMCs were prevented by the treatment with a ROS scavenger N-acetyl-cysteine (NAC), a NADPH oxidase inhibitor apocynin or knockdown of NOX2 with siRNA (Fig. 5B–D). Importantly, incubation of VSMCs with NAC, apocynin or NOX2 siRNA abolished the salusin-β-induced foam cell formation and adhesion of monocyte to VSMCs (Fig. 6B–G). Furthermore, salusin-β increased the ROS production, which were attenuated by NAC, apocynin or NOX2 siRNA (Fig. S9).

### miR155 is responsible for effects of salusin-β

**Figure 7:**
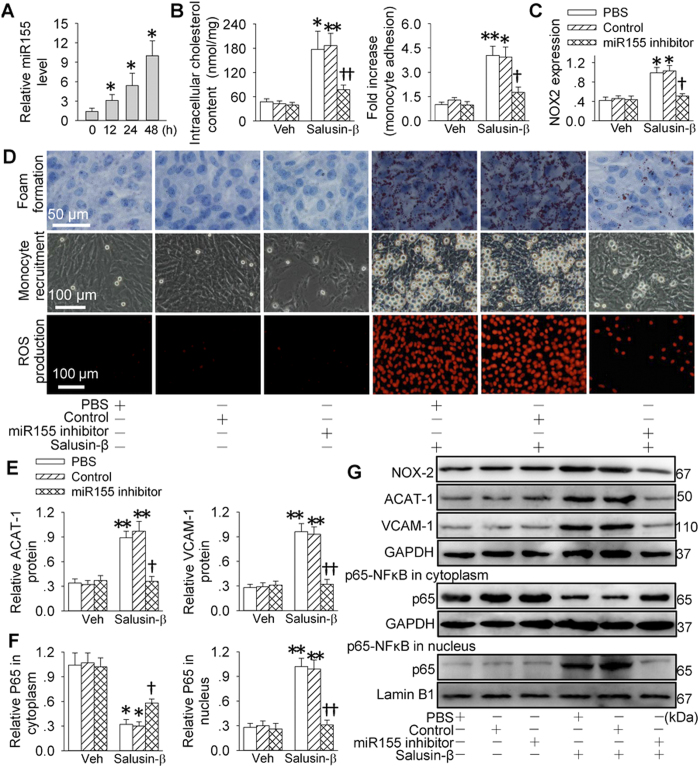
miR155 is responsible for salusin-β-induced foam formation, monocyte adhesion, NOX2 expression, ROS production, p65-NFκB nuclear translocation, and ACAT-1 and VCAM-1 expressions in VSMCs. (**A**) time-effects of salusin-β (30 nM) on miR155 expression; (**B**) effects of miR155 inhibitor on salusin-β-induced intracellular cholesterol content and monocyte adhesion. (**C**) effects of miR155 inhibitor on salusin-β-induced NOX2 expression; (**D**) effects of miR155 inhibitor on salusin-β-induced foam formation and monocyte recruitment; (**E**) effects of miR155 inhibitor on salusin-β-induced ACAT-1 and VCAM-1 expressions; (**F**) effects of miR155 inhibitor on salusin-β-induced p65-NFκB nuclear translocation. (**G**) representative images of Western blotting. In B-G, VSMCs were pretreated with miR155 inhibitor (100 nM) for 24 h and then stimulated with salusin-β (30 nM) for 48 h. Values are mean ± S.E.M. ^*^P < 0.05 and ^**^P < 0.01 vs. 0h or PBS. ^†^P < 0.05 and ^††^P < 0.01 vs. PBS+Salusin-β or Veh+Salusin-β.; n = 4 for each group.

Salusin-β time-relatedly increased miR155 expression in VSMCs and the maximal effect was observed at 48 hours after salusin-β treatment (Fig. 7A). Treatment with miR155 inhibitor in VSMCs prevented salusin-β-induced foam cell formation, monocyte adhesion to VSMCs, ROS production (Fig. 7B,D) and NOX2 expression (Fig. 7C,G). Furthermore, salusin-β-induced ACAT-1 and VCAM-1 expressions, and p65-NFκB nuclear translocation were diminished by miR155 inhibitor transfection in VSMCs (Fig. 7E–G). The efficiency of miR155 inhibitor was confirmed by the downregulation of miR155 levels in VSMCs (Fig. S10).

## Discussion

Atherosclerosis is a crucial underlying pathology of cardiovascular diseases. Foam cell formation is a major hallmark of early stage atherosclerotic lesions^19^. ACAT-1 is a key enzyme for intracellular cholesteryl ester synthesis^18^. Excessive cholesterol esterification resulted in accumulation of cholesterol ester stored as cytoplasmic lipid droplets and subsequently trigger the formation of foam cells^19^. Foam cells are not only derived from macrophages, but also from VSMCs^4^. It is known that circulating salusin-β levels is increased in coronary artery disease^20^ and salusin-β increased ACAT-1 expression in human monocyte-derived macrophages^15^. We found that salusin-β increased the accumulation of lipid droplets and intracellular total cholesterol content indicating that salusin-β promoted VSMCs-derived foam cell formation. In support of this notion, we found that ACAT-1 mRNA and protein expressions as well as the promoter activity in the ACAT-1 were upregulated in salusin-β-treated VSMCs, and knockdown of ACAT-1 with siRNA diminished salusin-β-induced increases in lipid droplets and intracellular total cholesterol contents in VSMCs. The results indicate the importance of ACAT-1 in salusin-β-induced foam cell formation from VSMCs.

Recruitments of monocytes and monocyte-derived phagocytes into the wall of large arteries accelerate the chronic inflammation and atherosclerosis^21^. ICAM-1 and VCAM-1 are important cell adhesion molecules and are merged as functional mediators in monocyte extravasation^22^. VCAM-1 expression is found in VSMCs, which promotes the migration of monocytes and lymphocytes into the vessel wall^23^. We found that salusin-β promoted the monocyte adhesion to VSMCs, and increased VCAM-1 mRNA and protein expressions as well as the promoter activity in the VCAM-1 in VSMCs. Knockdown of VCAM-1 with siRNA prevented the salusin-β-induced monocyte adhesion. These results indicate that salusin-β promotes monocyte recruitment to VSMCs and VCAM-1 is pivotal for the monocyte recruitment in VSMCs.

Transcription factor NFκB is implicated in inflammatory activation associated with the onset of atherosclerosis^24^. Translocation of p65 of NFκB from cytoplasm to nucleus is recognized as a prerequisite for the transcription^25^. VCAM-1 and ACAT-1 expressions at sites of atherosclerotic lesion formation are majorly controlled by NFκB system in early atherosclerotic lesions^26^. We found that salusin-β induced p65-NFκB nuclear translocation and recruitment of p65 to promoters of ACAT-1 and VCAM-1 in VSMCs. Inhibition of NFκB suppressed the salusin-β-induced ACAT-1 and VCAM-1 expressions, lipid accumulation in VSMCs and monocyte adhesion to VSMCs. These results indicate that p65-NFκB nucleus translocation is necessary for salusin-β-induced ACAT-1 and VCAM-1 expressions and the following foam cell formation from VSMCs and monocyte recruitment to VSMCs.

ROS is a critical contributor in the initiation and progression of atherosclerosis^27^. NFκB is a redox-sensitive transcription factor and can be regulated by intracellular redox status^28^. A major source for ROS in cardiovascular system is a family of NOX^29^,^30^. Our recent studies have shown that NAD(P)H oxidase-derived ROS in the brain is responsible for salusin-β-induced sympathetic activation^12^,^13^. In the present study, we found that salusin-β increased NOX2 expression and ROS production in VSMCs. Scavenging ROS, inhibiting NADPH oxidase or downregulating NOX2 abolished salusin-β-induced p65-NFκB nuclear translocation, ACAT-1 and VCAM-1 expressions, foam cell formation and monocyte adhesion in VSMCs. These results indicate that NOX2-derived ROS is a major downstream mediator of salusin-β in the ROS production and the subsequent p65-NFκB nuclear translocation, ACAT-1 and VCAM-1 expressions, foam cell formation and monocyte adhesion in VSMCs. MicroRNAs are small RNA molecules that regulate gene expression at post-transcriptional level^31^. MicroRNA-155 (miR155) is dominantly expressed in the atherosclerotic plaques and proinflammatory macrophages, and loss of miR155 reduces the proinflammatory NF-κB signaling associated with decreased recruitment of monocytes to atherosclerotic plaques^32^. Deficiency of miR155 attenuates atherogenesis in apolipoprotein E-deficient mice^33^. We found that salusin-β up-regulated miR155 level in VSMCs. Knockdown of miR155 abolished the salusin-β-induced NOX2 upregulation, ROS production, p65-NFκB nuclear translocation, ACAT-1 and VCAM-1 expressions, foam cell formation and monocyte adhesion in VSMCs. These results indicate that miR155 is crucial in salusin-β-induced oxidative stress and subsequent cascade process. A limitation in the present study was that the results were not examined *in vivo*, which need further investigation.

In conclusion, salusin-β upregulates miR155 in VSMCs, which increases NOX2-derived ROS production and subsequent nucleus translocation of p65-NFκB. The activation of NFκB promotes ACAT-1 and VCAM-1 expressions, and then causes foam cell formation from VSMCs and monocyte adhesion to VSMCs. Intervention of salusin-β may be a promising strategy for retarding the vascular lesions in atherosclerosis.

## Methods

### Cell culture

Human aortic VSMCs were obtained from American Type Culture Collection (Rockville, MD, USA). VSMCs were cultured as we previously reported^14^. In brief, VSMCs were maintained F12K Kaighn’s modification medium containing 10% fetal bovine serum (FBS) supplemented with 1× penicillin and streptomycin in a humidified cell incubator with standard cell culture condition (37 °C at 5% CO_2_ humidified atmosphere). Cells between passages 3 and 8 were used for the experiments. When cells reached 70–80% confluence, the cells were starved for 24 h in serum-free medium prior to use^34^.

### Oil red O staining

Oil red O staining for VSMCs was conducted according to previous study^35^. Simply, cultured VSMCs were plated on the 24-well plates and reached to 70–80% confluence, and then incubated with or without different doses of salusin-β for 12, 24 or 48 h in serum-free media. Afterwards, VSMCs were washed with 0.01 M PBS for three times, and stained with Oil Red O and Harris’ hematoxylin. The images were captured under a microscope at ×400 magnification.

### Intracellular total cholesterol measurement

Intracellular total cholesterol level was determined by enzymatic assay (Applygen Technologies, Beijing, China). Briefly, VSMCs were harvested into a centrifuge tube and then washed with PBS three times. Isopropylalcohol was employed to extract the intracellular lipids by ultrasonication. After centrifugation for 5 min at 2,000 g, the supernatant was used to determine the total cholesterol. The total cellular protein levels were quantified by Bradford assay method. The results in each sample were expressed in nmol of cholesterol per milligram of cellular protein and finally normalized to those of the control^36^.

### *In vitro* monocyte adhesion assay

Monocyte-VSMC binding assays were carried out as described previously^37^. U937 cells (monocytes) were purchased from FuDan IBS Cell Center (Shanghai, China) and cultured in RPMI-1640 Medium supplemented with 10% FBS. The VSMCs were incubated with or without salusin-β for 12, 24 or 48 h, respectively and followed by two rinses with PBS. Then, U937 cells (1 × 10^6^ cells/well) were added to VSMC culture and incubated for 1 h at 10 rpm at 37 °C. The medium was then drained and washed twice with FBS for removing the non-attached cells, the VSMC layers with attached monocytes were fixed with 4% paraformaldehyde and measured with a microscope at ×200 filed. The number of attached monocyte was randomly counted in five areas per well from three independent experiments and averaged.

### siRNA transfection

Human acyl coenzyme A:cholesterol acyltransferase-1 (ACAT-1) siRNA or human VCAM-1 siRNA (Santa Cruz, CA, USA) was transfected to human VSMCs as previously described^38^,^39^. Recommended scrambled siRNA (Santa Cruz, CA, USA) was used as a negative control. The human VSMCs were transfected with 100 nM of indicated siRNA with Lipofectamine 2000 reagent (Invitrogen, Carlsbad, CA, USA) for 24 h, the cells were plated for foam formation and monocyte adhesion assay after the 48 h of salusin-β treatment.

### Reporter gene transfection and luciferase activity assay

The forms of human VCAM-1 luciferase plasmids spanning −1350 to +45 bp or the core regions (−125 to +34) of human ACAT-1 proximal promoter was cloned into pGL3 basic luciferase reporter vector (Promega, WI, USA) as previous report^18^,^40^. VSMCs were transfected with 1 μg of luciferase plasmids or control pCMV-β-gal plasmid with lipofectamine 2000 (Invitrogen, Carlsbad, CA, USA) for 24 h. VSMCs were harvested and luciferase activity was measured using a dual luciferase reported gene assay kit (Beyotime Biotechnology, Shanghai, China). The luciferase activity was normalized to the internal control.

### Western Blot

VSMCs were lysed in lysis buffer and incubated for 30 min on the ice, the supernatant was retained after centrifugation. The total protein concentration in the supernatant was quantified with the Bradford assay (Santa Cruz, CA, USA). Equal amounts of protein in each sample were separated by SDS-PAGE electrophoresis and transferred to PVDF membrane. The membrane was blocked with 5% milk blocking buffer and incubated with the designed primary antibodies overnight at 4 °C. Horseradish peroxidase-conjugated secondary antibodies were used for detection. The protein bands were visualized with Enhanced Chemiluminescence Detection Kit (Thermo Scientific, Rockford, IL, USA).

### Real-time PCR

Total RNA was extracted with Trizol reagent following the manufacturer’s instructions. The cDNAs of human ACAT-1 and VCAM-1 were reversed with the PrimeScript^®^ RT reagent Kit and subjected to PCR amplification with SYBR Premix Ex Taq TM (Takara, Otsu, Shiga, Japan) on a StepOnePlus system (Applied Biosystems, Foster City, CA, USA). GAPDH was served as an internal control for mRNAs. Measurement of miR155 was conducted as previously reported^41^,^42^. Simply, the miR155 cDNAs were synthesized with Hairpin-it^TM^ miRNAs RT-PCR Quantitation kit (GenePharma, Shanghai, China) following the manufacturer’s protocols, and then real-time PCR was performed to determine the miR155 levels. U6 was served as an internal control for miR155. QPCR data was analyzed with cycle thresholds (Ct) methods and relative abundance of mRNA was normalized to the value of the house-keeping gene. The sequence-specific primers used were as follows. Human ACAT-1: 5′-TTCGGAATATCAAACAGGAGCC-3′ (forward), 5′-CACACCTGGCAAGATGGAGTT-3′ (reverse); human VCAM-1: 5′-CCTGCCATTGGAATGATAA-3′ (forward), 5′-TGCTTCTACAAGACTATATGAC-3′ (reverse); human GAPDH: 5′-TGTTGCCATCAATGACCCCTT-3′ (forward), 5′-CTCCACGACGTACTCAGCG-3′ (reverse). miR155: 5-GCTTCGGTTAATGCTAATCGTG-3 (forward), 5-CAGAGCAGGGTCCGAGGTA-3 (reverse); U6: 5-CTCGCTTCGGCAGCACA-3 (sense), 5-AACGCTTCACGAAYY YGCGT-3 (reverse)^15^.

### Preparation of cytoplasmic fractions and nuclear extracts

Nuclear and cytoplasmic extracts were prepared and obtained as previous report^43^. The purity of nuclear extracts was evaluated by immunoblotting with incubation of primary antibodies against lamin B1 (Santa Cruz, CA, USA). The purity of cytoplasmic fractions was confirmed by immunoblotting with primary antibodies against GAPDH (Santa Cruz Biotechnology, CA, USA).

### Chromatin immunoprecipitation (ChIP)

ChIP assays were conducted as described previously^44^. The ChIP-enriched DNA fragments were immunoprecipitated in the presence or absence of p65 and Pol II antibodies, and a rabbit monoclonal control IgG (Santa Cruz Biotechnology, CA, USA). qPCR was used to assess the NFκB and Pol II binding site on the ACAT-1 and VCAM-1 promoters. The data were quantified and normalized with input samples and expressed as fold change of control. Precipitated DNA fragments were amplified by PCR. The primers encompassing the p65 site were as follows. In ACAT-1 promoter: 5′-CTTAACCTGGGGACCACCAATAG-3′ (forward), 5′-CTTCTATTGGTGGTCCCCAGGTT-3 (reverse)^40^; in VCAM-1 promoter: 5′-AAATCAATTCACATGGCATA-3′ (forward), 5′-AAGGGTCTTGTTGCAGAGG-3′ (reverse)^45^.

### Lentiviral vector transduction in VSMCs

VSMCs were seeded at 10^5 ^cells/well in 6-well plates 24 h prior to transfection. The VSMCs were grown to a 40–60% confluence and transfected with recombinant lentivirus vector harboring ACAT-1 (sc-402300-LAC, Santa Cruz, CA, USA) or VCAM-1 (sc-400135-LAC, Santa Cruz, CA, USA) in serum-free growth medium overnight. The viruses contained medium was replaced with 3 ml of standard medium. Measurements were conducted 2 days after transfection^46^.

### Measurement of ROS generation

DHE fluorescent dye was used to determine intracellular superoxide anion generation in VSMCs as previous report^47^. VSMCs were fixed and loaded with DHE (10 μmol/L) for 30 min in a light-protected humidified chamber. The fluorescence (488/525 nm) were obtained with fluorescence microscope.

### Chemicals and antibodies

Human salusin-β was obtained from Phoenix Pharmaceuticals (Belmont, CA, USA). Cell culture supplies were purchased from Costar (Corning Inc., Cypress, CA, USA). Antibodies against ACAT-1, VCAM-1, GAPDH, Lamin B1 and indicated horseradish peroxidase (HRP) conjugated secondary antibodies were purchased from Santa Cruz Biotechnology Co. (Santa Cruz, CA, USA). Antibody against p65 was obtained from Cell Signaling Technology (Beverly, MA, USA). Antibody against NOX2 was purchased from Abcam (Cambridge, MA, USA). Sequence of human miR155 inhibitor is 5′-ACCCCUAUCACAAUUAGCAUUAA-3′ and the sequence of its control is 5′-CAGUACUUUUGUGUAGUACAA-3′ (GenePharma, Shanghai, China). Bay11-7082, N-acetyl-L-cysteine (NAC), deihydroethidium and the reporter plasmid pGL6-NFκB-Luc for measurement of NFκB activity were obtained from Beyotime Biotechnology (Shanghai, China). Apocynin and dimethyl sulfoxide (DMSO) were obtained from Sigma Chemical (St. Louis, MO, USA).

### Statistical analysis

Data were expressed as mean ± S.E.M. One-way or two-way ANOVA followed by post hoc Bonferroni test was used for multiple comparisons. A value of *P* < 0.05 was considered statistically significant.

## Additional Information

**How to cite this article**: Sun, H.-J. *et al*. Salusin-β induces foam cell formation and monocyte adhesion in human vascular smooth muscle cells via miR155/NOX2/NFκB pathway. *Sci. Rep.* **6**, 23596; doi: 10.1038/srep23596 (2016).

## Supplementary Material

Supplementary Information
