# Supplementary material for: Salusin-β induces foam cell formation and monocyte adhesion in human vascular smooth muscle cells via miR155/NOX2/NFκB pathway
Source: Sci Rep. 2016 Mar 23;6:23596. doi: 10.1038/srep23596 (PMC4804242; doi:10.1038/srep23596)
Supplement: Supplementary Information [file srep23596-s1.doc]

**Salusin-β induces foam cell formation and monocyte adhesion in human vascular smooth muscle cells via miR155/NOX2/NFκB pathway**

Hai-Jian Sun^a^, Ming-Xia Zhao^a^, Tong-Yan Liu^a^, Xing-Sheng Ren^a^, Qi Chen^b^, Yue-Hua Li^b^, Yu-Ming Kang^c^, Guo-Qing Zhu^a,b^*****

^a^ Key Laboratory of Cardiovascular Disease and Molecular Intervention, Department of Physiology, Nanjing Medical University, Nanjing, Jiangsu 210029, China; ^b^ Department of Pathophysiology, Nanjing Medical University, Nanjing, Jiangsu 210029, China; ^c^ Department of Physiology and Pathophysiology, Cardiovascular Research Center, Xi'an Jiaotong University School of Medicine, Xi'an 710061, China

***Address for correspondence:**

Guo-Qing Zhu, M.D., Ph.D. Professor, Chair

Key Laboratory of Cardiovascular Disease and Molecular Intervention, Department of Physiology, Nanjing Medical University, 140 Hanzhong Road, Nanjing 210029, China.

Tel: +86-25-86862885; Fax: +86-25-86862885; E-Mail: [gqzhucn@njmu.edu.cn](mailto:gqzhucn@njmu.edu.cn)

**Supplemental Figures**


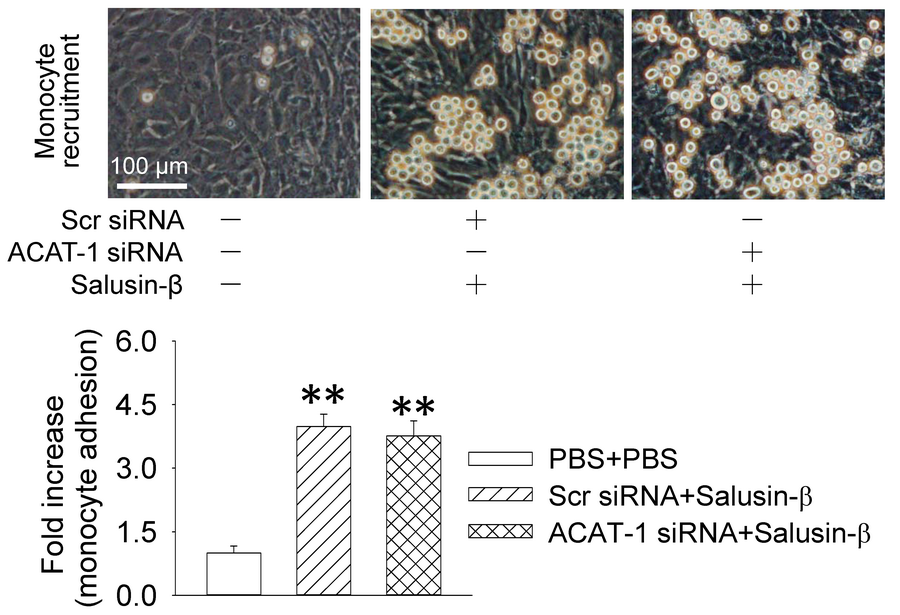


Figure S1. Effects of knockdown of ACAT-1 on monocyte recruitment responses to salusin-β in VSMCs. VSMCs were transfected with ACAT-1 siRNA or scrambled (Scr) siRNA (100 nM) for 24 h, and then treated with or without salusin-β (30 nM) for 48 h. Values are mean±S.E.M. ** P<0.01 vs. PBS+PBS. n=4 for each group.


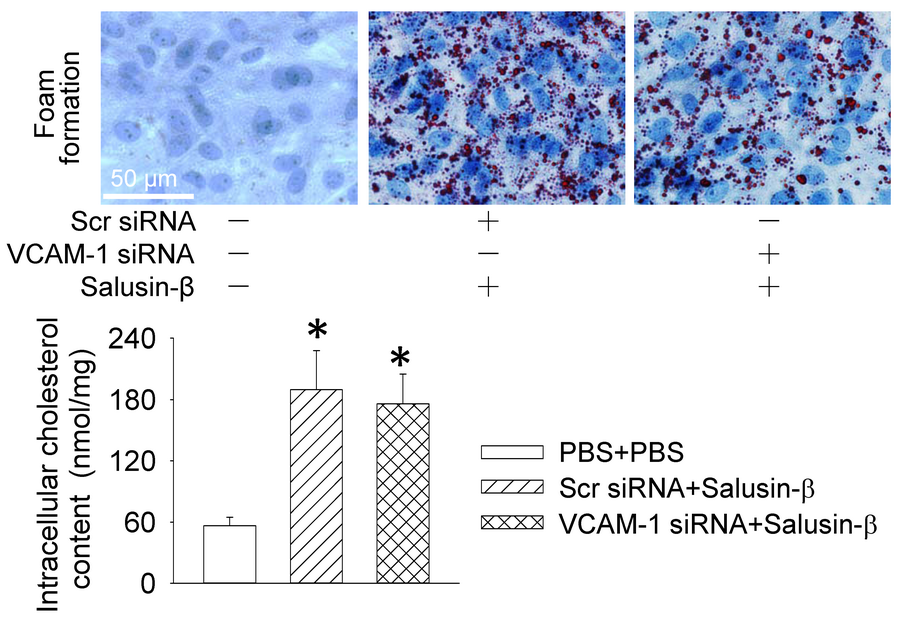


Figure S2. Effects of knockdown of VCAM-1 on foam formation and intracellular cholesterol content responses to salusin-β in VSMCs. VSMCs were transfected with VCAM-1 siRNA or scrambled (Scr) siRNA (100 nM) for 24 h, and then treated with or without salusin-β (30 nM) for 48 h. Values are mean±S.E.M. *P<0.05 vs. PBS+PBS. n=6 for each group.


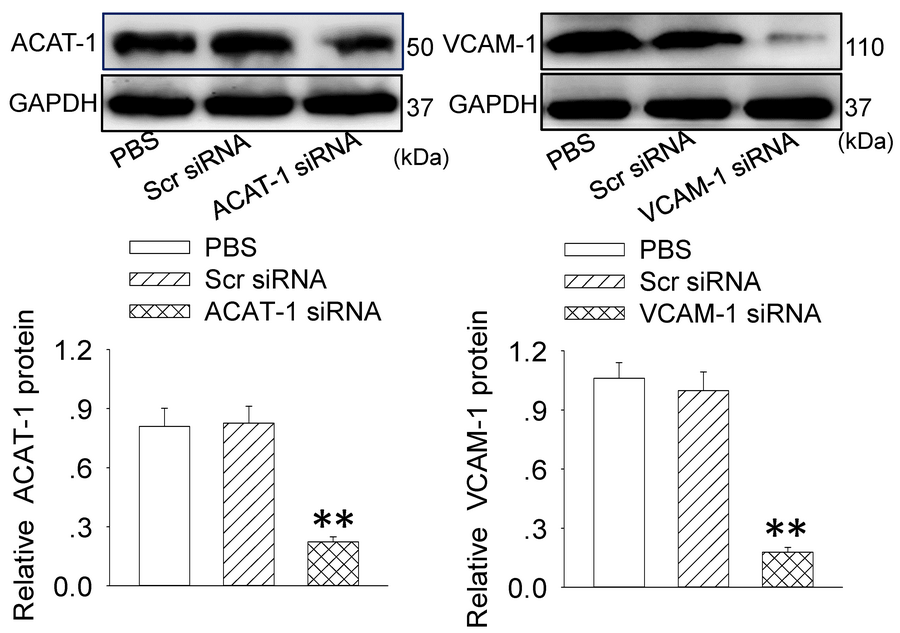


Figure S3. Effect of knockdown of ACAT-1 and VCAM-1 on the expressions of ACAT-1 and VCAM-1 responses to salusin-β in VSMCs. VSMCs were transfected with VCAM-1 siRNA or scrambled (Scr) siRNA (100 nM) for 24 h, and then treated with salusin-β (30 nM) for 48 h. Values are mean±S.E.M. ** P<0.01 vs. Veh or Scr siRNA. n=4 for other groups.


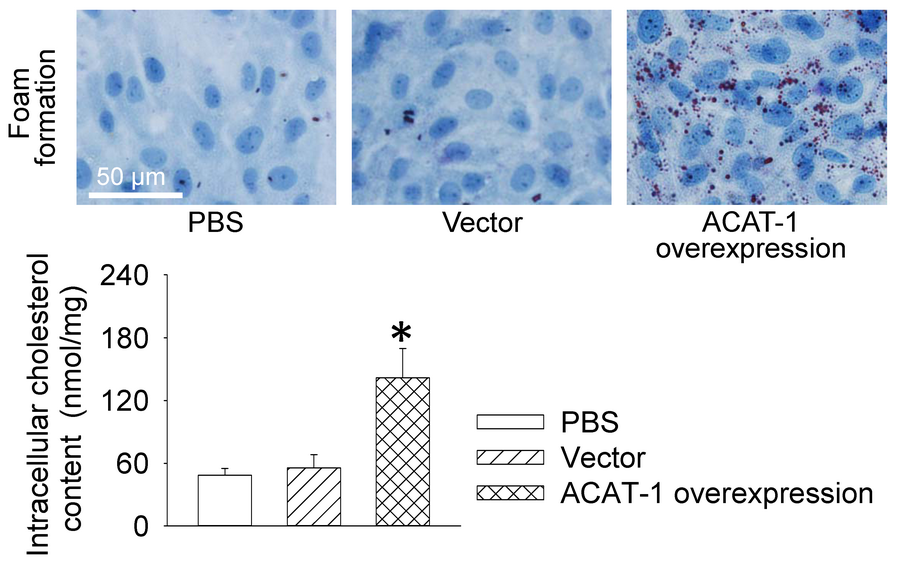


Figure S4. Effects of ACAT-1 overexpression on foam formation and intracellular cholesterol content in VSMCs. VSMCs were transfected with lentivirus-mediated ACAT-1 (10 μl/ml) for 48 h. Values are mean±S.E.M. *P<0.05 vs. PBS or Vector. n=6 for each group.


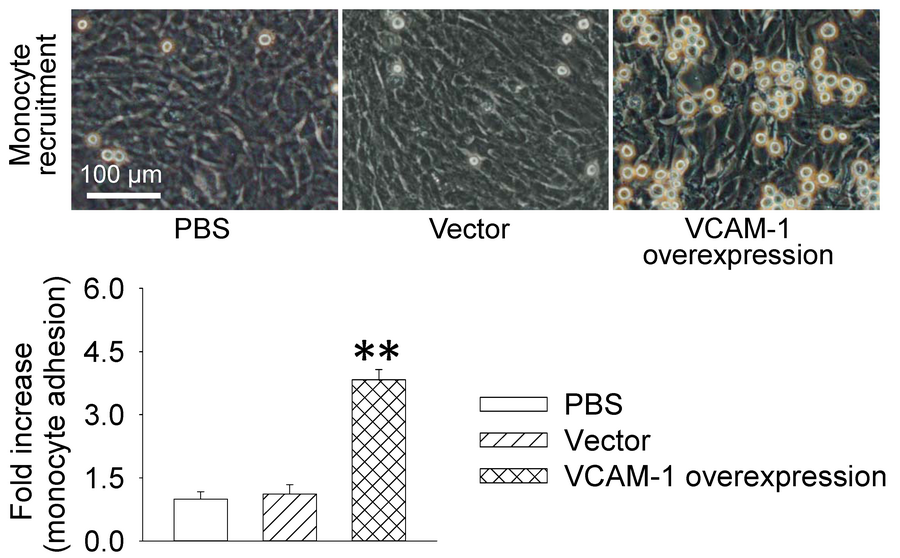


Figure S5. Effects of VCAM-1 overexpression on monocyte recruitment in VSMCs. VSMCs were transfected with lentivirus-mediated VCAM-1 (10 μl/ml) for 48 h. Values are mean±S.E.M. **P<0.01 vs. PBS or Vector. n=6 for each group.


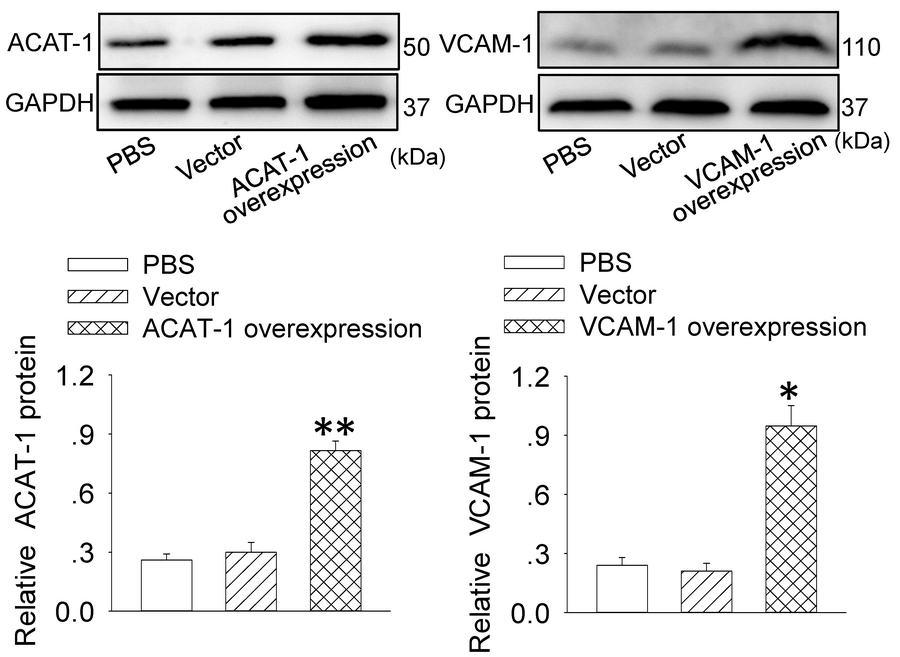


Figure S6. Effect of ACAT-1 and VCAM-1 overexpression on ACAT-1 and VCAM-1 protein levels in VSMCs. VSMCs were transfected with lentivirus-mediated ACAT-1 (10 μl/ml) or VCAM-1 (10 μl/ml) for 48 h. Values are mean±S.E.M. * P<0.05 and ** P<0.01 vs. Veh. n=4 for each group.


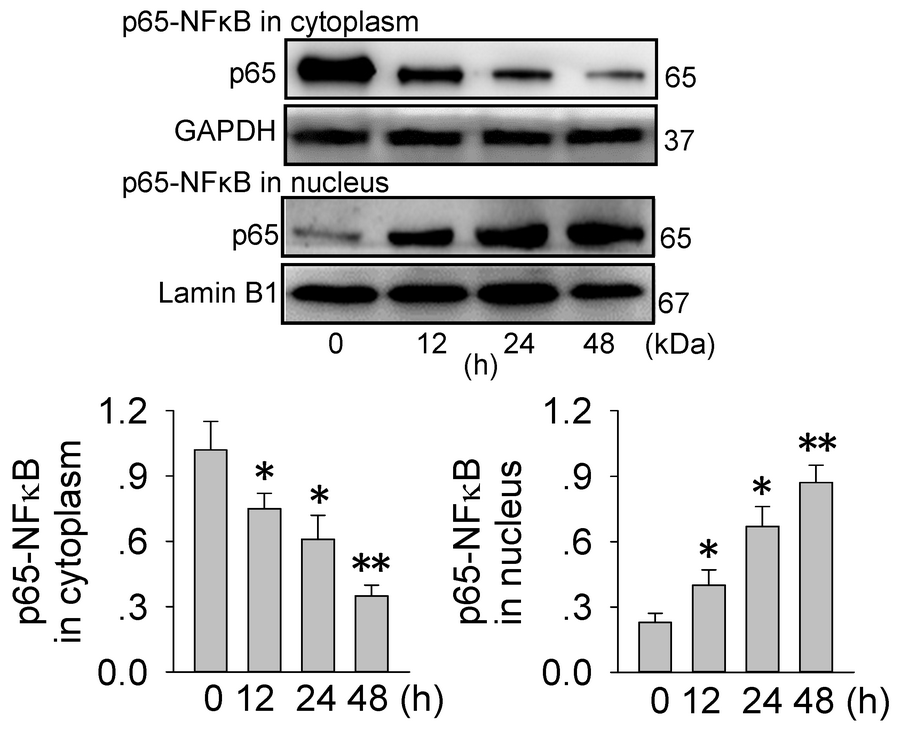


Figure S7. Time effects of salusin-β (30 nM) on p65-NFκB nuclear translocation in VSMCs. Values are mean±S.E.M. *P<0.05 and ** P<0.01 vs. 0 h. n=4 for each group.


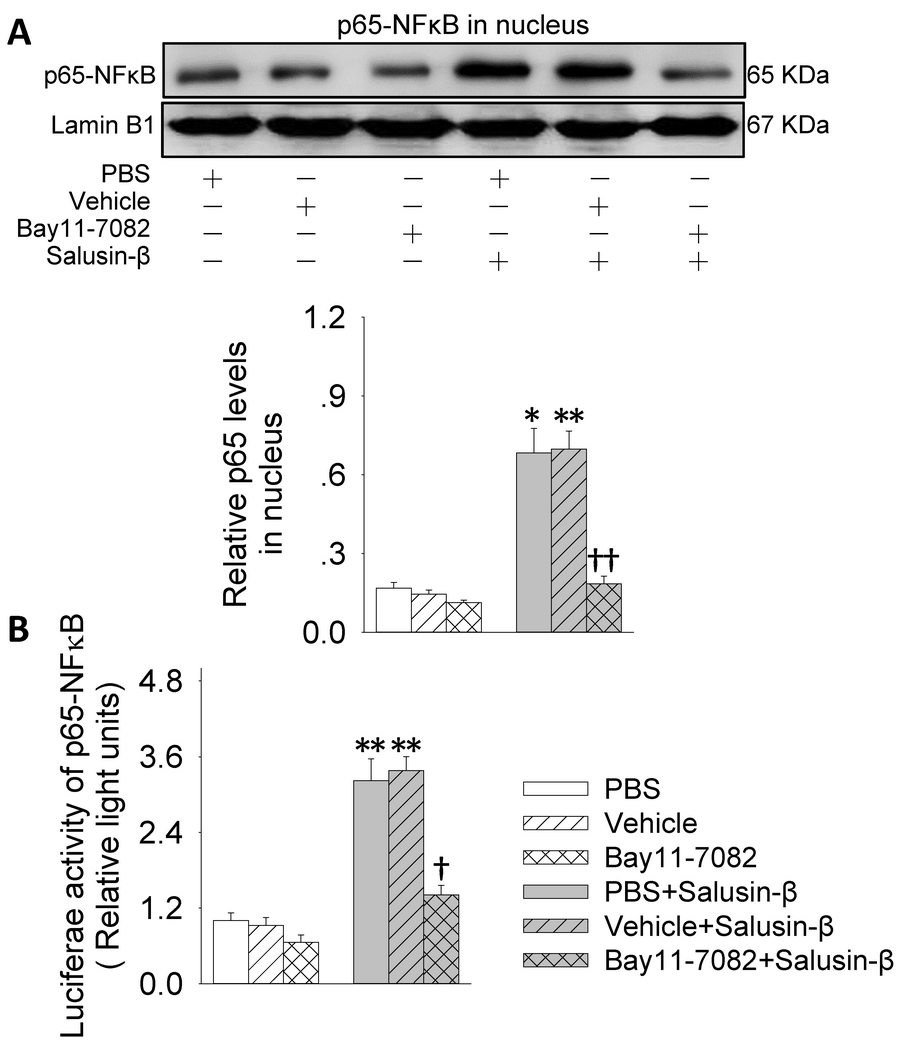


Figure S8. Effects of a NFκB inhibitor Bay11-7082 on the salusin-β-induced changes in nuclear NFκB levels and NFκB activity in VSMCs. A, effects of Bay11-7082 on nuclear p65-NFκB levels. VSMCs were incubated with Bay11-7082 for 6 h and then treated with or without salusin-β (30 nM) for 48 h. B, effects of Bay11-7082 on promoter activity of p65-NFκB. VSMCs were transiently co-transfected with 1μg of pGL6-p65-NFκB Luc plasmid or control pRL-TK plasmid with Lipofectamine 2000. After transfection for 6 h, cells were pretreated with or without Bay11-7082 and then challenged by salusin-β (30 nM) for additional 48 h. Luciferase activity was normalized to the activity of the internal control. Values are mean±S.E.M. *P<0.05 and ** P<0.01 vs. PBS or Vehicle. †P<0.05 and †† P<0.01 vs. PBS+Salusin-β or vehicle+Salusin-β. n=4 for each group.


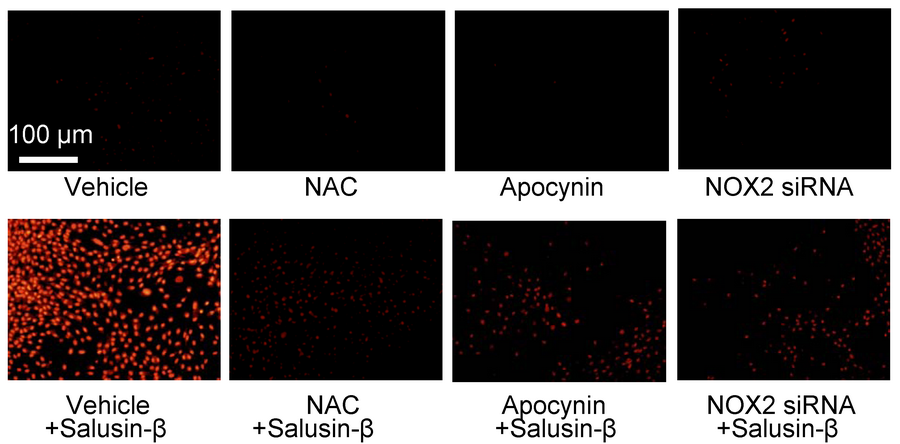


Figure S9. Effects of pretreatment with NAC, apocynin or NOX2 siRNA on ROS production response to salusin-β in VSMCs. VSMCs were incubated with NAC, apocynin or NOX2 siRNA for 8 h, and then stimulated with salusin-β (30 nM) for 48 h. n=4 for each group.


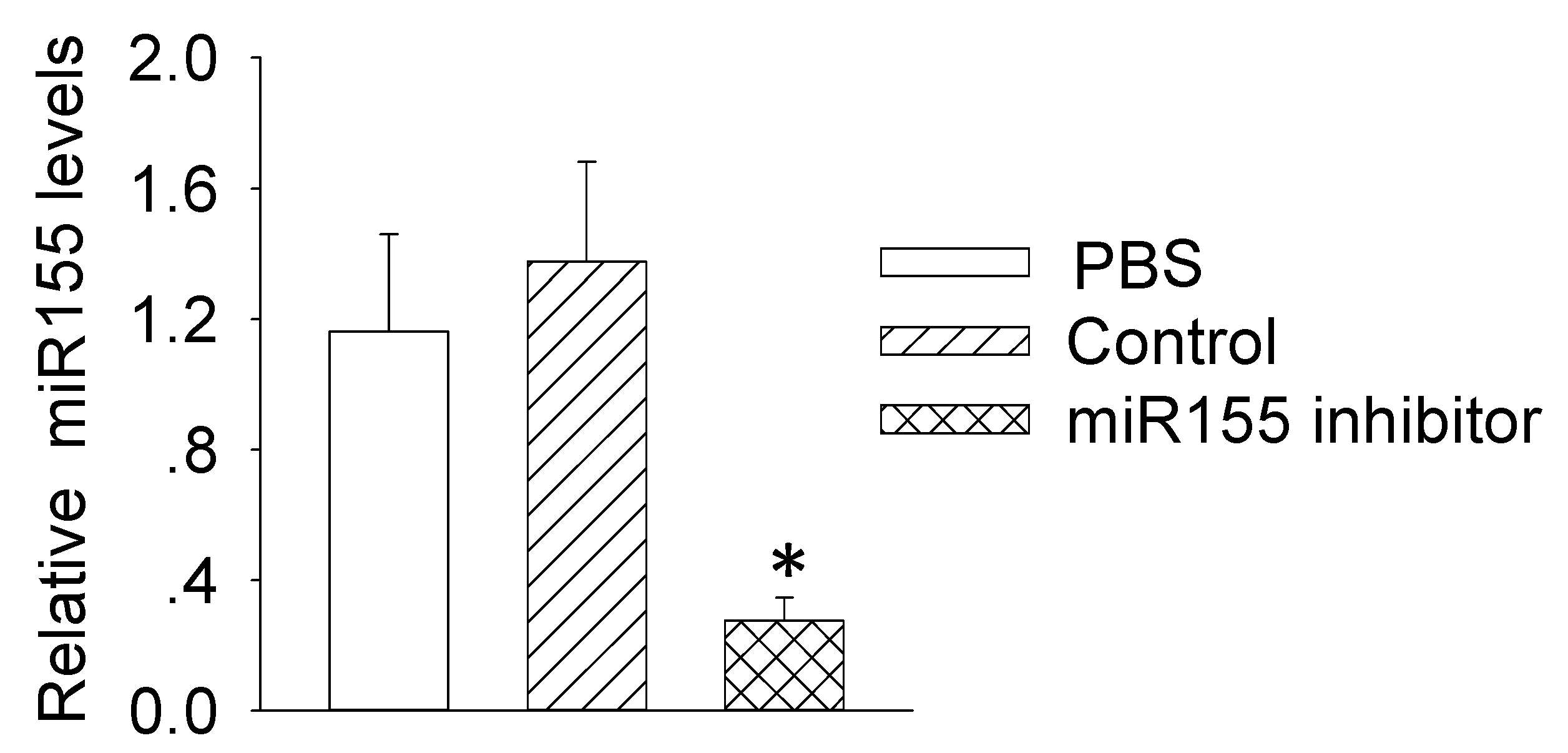


Figure S10. Effects of miR155 inhibitor on miR155 levels in VSMCs. VSMCs were incubated with miR155 inhibitor (100 nM) for 24 h. *P<0.05 vs. PBS or Control. n=6 for each group.
